# Editorial for the Special Issue on Optical MEMS

**DOI:** 10.3390/mi10070458

**Published:** 2019-07-07

**Authors:** Huikai Xie, Frederic Zamkotsian

**Affiliations:** 1 Department of Electrical and Computer Engineering, University of Florida, Gainesville, FL 32611, USA; 2 Aix Marseille Univ, CNRS, CNES, LAM, Laboratoire d’Astrophysique de Marseille, 38 rue Frederic Joliot Curie, 13388 Marseille CEDEX 13, France

Optical micro-electro-mechanical systems (MEMS), micro-opto-electro-mechanical systems (MOEMS), or optical microsystems are devices or systems that interact with light through actuation or sensing at a micron or millimeter scale. Optical MEMS have had enormous commercial success in projectors, displays, and fiber optic communications. The best known example is Texas Instruments’ digital micromirror devices (DMDs). The development of optical MEMS was impeded seriously by the Telecom Bubble in 2000. Fortunately, DMDs grew their market size even in that economy downturn. Meanwhile, in the last one and half decades, the optical MEMS market has been slowly but steadily recovering. During this time span, the major technological change was the shift of thin-film polysilicon microstructures to single-crystal-silicon microstructures. Especially in the last few years, cloud data centers demand large-port optical cross connects (OXCs), autonomous driving looks for miniature light detection and ranging systems (LiDAR), and virtual reality/augmented reality (VR/AR) demands tiny optical scanners. This is a new wave of opportunities for optical MEMS. Furthermore, several research institutes around the world have been developing MOEMS devices for extreme applications (very fine tailoring of light beam in terms of phase, intensity, or wavelength) and/or extreme environments (high vacuum or cryogenic temperature) for many years.

This special issue contains twelve research papers covering MEMS mirrors [1,2,3,4,5,6,7,8,9,10], MEMS variable optical attenuators (VOAs) [11], and tunable spectral filters [12]. These MEMS devices are based on three of the commonly used actuation mechanisms: electrothermal [1], electrostatic [2,3,4,5,6,7,11], and electromagnetic actuation [8,9,10]. MEMS optical scanners involving single mirrors are demonstrated or used in [1,2,3,8,9,10], while all other optical microsystems employ MEMS mirror arrays that are all based on DMDs [4,5,6,7]. This special issue also includes one review paper on metalens-based miniaturized optical systems [13]. 

Among the papers on single MEMS mirrors, two are focused on MEMS device fabrication [1,10], one on optimization of the driving signals [3], one on applying MEMS mirrors for confocal microscopy [2], and two on using MEMS mirrors to generate structural light patterns for 3D measurement [8,9]. Interestingly, there are several papers reporting various applications of DMDs, including spectral filtering [4], Hadamard spectroscopy [5], wavefront/aberration correction [6], and a tunable fiber laser [7].

In particular, Zhou et al. presented the design, fabrication, and characterization of an electrothermal MEMS mirror with large tip-tilt scan around ±8° and large piston scan of 114 μm at only 2.35 V as well as large resonance frequencies of 1.5 kHz (piston) and 2.7 kHz (tip-tilt); this device survived 220 billion scanning cycles [1]. Lei et al. developed a low-cost FR4-based electromagnetic scanning micromirror integrated with an electromagnetic angle sensor; this MEMS mirror achieved an optical scan angle of 11.2° with a low driving voltage of only 425 mV at resonance (361.8 Hz) [10]. Kim et al. demonstrated an original driving scheme of an electrostatic microscanner in a quasi-static mode based on an input shaping method by an experimental transfer function; the usable scan range was extended up to 90% or higher for most frequencies up to 160 Hz [3].

On the applications side, Yao et al. modified a confocal microscope for including a resonant MEMS scanner in order to miniaturize the system [2]. Hu et al. proposed a new multiple laser stripe scanning profilometry based on a scanning mirror that can project high quality movable laser stripes, delivering high-quality images, mechanical movement noise elimination, and speckle noise reduction [5]. Yang at al. combined the high accuracy of the fringe projection profilometry with the robustness of the laser stripe scanning and demonstrated 3D shape measurement of surfaces with large reflection variations using a biaxial scanning micromirror projection system [9].

Gao et al. showed that a programmable filter based on a DMD can experimentally reach a minimum bandwidth as low as 12.5 GHz in C-band, where the number of channels and the center wavelength can be adjusted independently, as well as the channel bandwidth and the output power [4]. Lu et al. employed a new Hadamard mask of variable-width stripes to improve the Signal-to-Noise Ratio (SNR) of a Hadamard transform near-infrared spectrometer by reducing the influence of stray light [5]. Carmichael Martins et al. confirmed that using a DMD for aperture scanning can perform efficiently to measure ocular aberrations sequentially, even for highly aberrated wavefronts [6]. Li et al. demonstrated a tunable fiber laser with high tuning resolution in the C-band, based on a DMD chip as a programmable wavelength filter, and an echelle grating to achieve high-precision tuning [7].

Finally, Sun et al. were able to reduce the wavelength-dependent loss (WDL) and the polarization-dependent loss (PDL) of MEMS-based variable optical attenuators (VOAs) by using a specific shape of the end-face of the collimating lens [11]. Liu et al. have chosen to use a new dual-mode liquid-crystal (LC) device incorporating a Fabry–Perot cavity and an arrayed LC micro-lens for performing simultaneous electrically adjusted filtering and zooming in the infrared wavelength range by adjusting the transmission spectrum and the point spread function of the incident micro-beams [12]. Li et al. reviewed the use of a metasurface-based flat lens (metalens) for miniaturized optical imaging and sensing systems, especially in the bio-optics field, including a large field of view (FOV), chromatic aberration, and high-resolution imaging [13].

We would like to take this opportunity to thank all the authors for submitting their papers to this Special Issue. We also want to thank all the reviewers for dedicating their time and helping to improve the quality of the submitted papers.

## References

[1] Zhou L., Zhang X., Xie H. (2019). An Electrothermal Cu/W Bimorph Tip-Tilt-Piston MEMS Mirror with High Reliability. Micromachines.

[2] Yao C.Y., Li B., Qiu Z. (2019). 2D Au-Coated Resonant MEMS Scanner for NIR Fluorescence Intraoperative Confocal Microscope. Micromachines.

[3] Kim K., Moon S., Kim J., Park Y., Lee J.H. (2019). Input Shaping Based on an Experimental Transfer Function for an Electrostatic Microscanner in a Quasistatic Mode. Micromachines.

[4] Gao Y., Chen X., Chen G., Tan Z., Chen Q., Dai D., Zhang Q., Yu C. (2019). Programmable Spectral Filter in C-Band Based on Digital Micromirror Device. Micromachines.

[5] Lu Z., Zhang J., Liu H., Xu J., Li J. (2019). The Improvement on the Performance of DMD Hadamard Transform Near-Infrared Spectrometer by Double Filter Strategy and a New Hadamard Mask. Micromachines.

[6] Carmichael Martins A., Vohnsen B. (2019). Measuring Ocular Aberrations Sequentially Using a Digital Micromirror Device. Micromachines.

[7] Li J., Chen X., Dai D., Gao Y., Lv M., Chen G. (2019). Tunable Fiber Laser with High Tuning Resolution in C-band Based on Echelle Grating and DMD Chip. Micromachines.

[8] Hu G., Zhou X., Zhang G., Zhang C., Li D., Wang G. (2019). Multiple Laser Stripe Scanning Profilometry Based on Microelectromechanical Systems Scanning Mirror Projection. Micromachines.

[9] Yang T., Zhang G., Li H., Zhou X. (2019). Hybrid 3D Shape Measurement Using the MEMS Scanning Micromirror. Micromachines.

[10] Lei H., Wen Q., Yu F., Zhou Y., Wen Z. (2018). FR4-Based Electromagnetic Scanning Micromirror Integrated with Angle Sensor. Micromachines.

[11] Sun H., Zhou W., Zhang Z., Wan Z. (2018). A MEMS Variable Optical Attenuator with Ultra-Low Wavelength-Dependent Loss and Polarization-Dependent Loss. Micromachines.

[12] Liu Z., Chen M., Xin Z., Dai W., Han X., Zhang X., Wang H., Xie C. (2019). Research on a Dual-Mode Infrared Liquid-Crystal Device for Simultaneous Electrically Adjusted Filtering and Zooming. Micromachines.

[13] Li B., Piyawattanametha W., Qiu Z. (2019). Metalens-Based Miniaturized Optical Systems. Micromachines.

